# Sustainable Meat Alternatives: Incorporation of *Tenebrio molitor* and *Alphitobius diaperinus* Powders into Pork-Based Hybrid Hams

**DOI:** 10.3390/foods14071192

**Published:** 2025-03-28

**Authors:** Lisiane Carvalho, Adriana Ferreira, Ana Novo Barros, Maria Otília Carvalho, Teresa J. S. Matos, Anabela Raymundo, Isabel Sousa

**Affiliations:** 1 LEAF—Linking Landscape, Environment, Agriculture and Food Research Center, Instituto Superior de Agronomia, Universidade de Lisboa, Tapada da Ajuda, 1349-017 Lisboa, Portugal; motiliac@isa.ulisboa.pt (M.O.C.); matosteresa@isa.ulisboa.pt (T.J.S.M.); anabraymundo@isa.ulisboa.pt (A.R.); isabelsousa@isa.ulisboa.pt (I.S.); 2 R&D Departamento, Primor Charcutaria-Prima, S.A., Gavião, 4760-003 Vila Nova de Famalicão, Portugal; adriana.ferreira@primor.pt; 3 CITAB (Centre for the Research and Technology of Agro-Environment and Biological Sciences), Inov4Agro (Institute for Innovation, Capacity Building and Sustainability of Agri-Food Production), University of Trás-os-Montes and Alto Douro (UTAD), Quinta de Prados, 5000-801 Vila Real, Portugal; abarros@utad.pt

**Keywords:** consumer acceptance, edible insects, nutritional composition, meat substitutes

## Abstract

The increasing demand for sustainable meat alternatives has driven research into edible insects as a protein source. This study developed and characterized hybrid hams using pork meat with 10% of *Tenebrio molitor*, 10% of *Alphitobius diaperinus,* or 5% of *A. diaperinus* plus 5% of *T. molitor* powders. The hybrid hams were analyzed for color, texture, nutritional composition, amino acid profile, antioxidant activity, and consumer acceptance. Results indicated that adding insect powder led to a darker color in hybrid hams. The protein content increased, reaching 49% in the 10% *T. molitor* and 46% in the 10% *A. diaperinus* formulations, compared to 35% in the control (without insect powder). Amino acid analysis of the 10% *A. diaperinus* formulation revealed higher concentrations of essential amino acids compared to the control, with threonine increasing by 185%, valine by 24% and histidine by 27%. Also, the inclusion of insect powders enhanced the mineral profile, mainly sodium, potassium, phosphorus, and sulfur. The total polyphenol content nearly doubled in the 10% *A. diaperinus* and mixed formulations. Additionally, sensory evaluation revealed that these formulations were well-accepted. These findings support the potential of edible insects as a sustainable and nutritious protein source for innovative food products.

## 1. Introduction

The reduction of red meat consumption has become increasingly significant due to its implications for health, the environment, and animal welfare. Alternative meat products present an opportunity to guide people’s diets in a more sustainable direction. To foster greater acceptance and encourage the adoption of these alternatives, it is essential to identify the most appealing protein sources from the perspective of consumers [1,2].

Edible insects have received growing interest as a sustainable protein source mainly because of their environmental and nutritional advantages. Also, edible insect proteins can be used as functional food ingredients for providing technological benefits, such as emulsion capacity, gel-forming and water/oil absorption ability [3,4,5]. Thus, it is reasonable to accept that insect proteins can be used to partially substitute the meat portion in processed meat products without compromising the nutritional and technological properties [6].

In addition to their benefits as rich protein sources, studies have demonstrated that edible insects are abundant in vitamins, minerals, and essential amino acids. Moreover, the exploration of functional substances in edible insects, such as antimicrobial peptides and active polysaccharides, has opened new applications in food technology. These findings underscore the growing interest in and potential of edible insects as a valuable food source in the quest for sustainable and nutritious food sources [7,8].

In the European Union, edible insects are classified as a novel food, whereby their introduction to the European market is subject to Regulation (EU) 2015/2283. Regulation (EU) 2021/882 authorizes the placing on the market of dehydrated *Tenebrio molitor* larvae as a novel food, with a maximum limit of 10 g/100 g in powder form in protein products. Also, Regulation (EU) 2023/58 authorizes the placing on the market of the larvae of *Alphitobius diaperinus* as a new food, where a maximum of 15 g/100 g can be used in powder form and 40 g/100 g in frozen or paste form in meat analogues [9,10].

Additionally, Regulation (EU) 2021/1975 authorizes the placing on the market of frozen, dehydrated, and powdered forms of *Locusta migratoria* as a novel food under Regulation (EU) 2015/2283, with a maximum content of 80 g/100 g in frozen form and 50 g/100 g in dehydrated or powdered form for meat analogues. Similarly, Regulation (EU) 2022/188 authorizes the placing on the market of frozen, dehydrated, and powdered forms of *Acheta domesticus* under the same regulation, with the same content limits. Furthermore, on 20 January 2025, the Commission through Regulation 2025/89, authorized the placing on the market of UV-treated powder of whole *Tenebrio molitor* larvae as a novel food for use in various products such as bread and rolls, cakes, pasta-based products, processed potato products, cheese and cheese products, and fruit and vegetable preserves [11,12,13]. These regulations provide incentives to promote the development and consumption of insect-based foods.

Regarding insects as a food source, people may find it easier to consume insect-based foods rather than consuming whole insects [14] and their acceptance is influenced by cultural and demographic factors. A consumer survey carried out by Maciejewska et al. [15] revealed that Polish respondents, particularly younger individuals in urban areas, showed a greater willingness to incorporate these products into their diets. In contrast, Spanish consumers displayed more hesitation, likely due to deep-rooted dietary traditions and differences in food education. Emphasizing the nutritional and environmental benefits while incorporating insects into recognizable formats may facilitate broader acceptance, particularly among hesitant consumers.

*Tenebrio molitor* larvae, widely consumed globally, are recognized as a promising alternative to conventional food proteins due to their notable content of protein, lipids, vitamins and minerals, as well as offering both environmental and economic benefits. Their protein content ranges from 41% to 66% and is considered high-quality, with an amino acid profile rich in essential amino acids. Additionally, their lipid content, comprising 15% to 50%, is predominantly monounsaturated fatty acids. The larvae are also rich in minerals such as potassium, calcium, iron and magnesium, as well as vitamins like riboflavin (B2), pantothenic acid (B5), biotin (B7), B12, and C [16].

Compared to *Tenebrio molitor*, *Alphitobius diaperinus* has attracted little interest, probably because of the small size of its larvae and the limited technological knowledge about their rearing. However, it has a faster development cycle, better reproduction rate and softer exoskeleton than similar edible beetle larvae. For human consumption, raw larvae of *A. diaperinus* can be roasted or used in a hidden form after grinding, with the latter use being more acceptable. For inclusion of *A. diaperinus* in food, a detailed knowledge about its chemical composition is essential. The nutritional value of insects depends on many factors, such as feed, temperature and time of harvest. Although *A. diaperinus* appear to be good sources of protein (58%) and lipids (26%), data on this topic is limited [17,18,19].

In summary, both *Tenebrio molitor* and *Alphitobius diaperinus* are highly nutritious and safe for consumption according to Regulations (EU) 2021/882 and 2023/58, respectively. However, these regulations require labeling to indicate that these ingredients may cause allergic reactions in individuals with known allergies to crustaceans, their derivatives, and dust mites [9,10]. Additionally, research highlights the potential of *Tenebrio molitor* as a functional ingredient in the food industry due to its bioactive properties, including emulsification, water and oil absorption capacity and antioxidant activity. Similarly, *Alphitobius diaperinus* is recognized for its antioxidant and antihypertensive activities, as well as its hydration behavior, making it a valuable functional ingredient in various food applications [15,20,21,22].

Based on these findings, the objective of this study was to develop and characterize hybrid hams using pork meat with 10% of *Tenebrio molitor*, 10% of *Alphitobius diaperinus*, or 5% of *A. diaperinus* plus 5% of *T. molitor* powders.

## 2. Materials and Methods

### 2.1. Hybrid Hams Preparation

**Figure 1:**
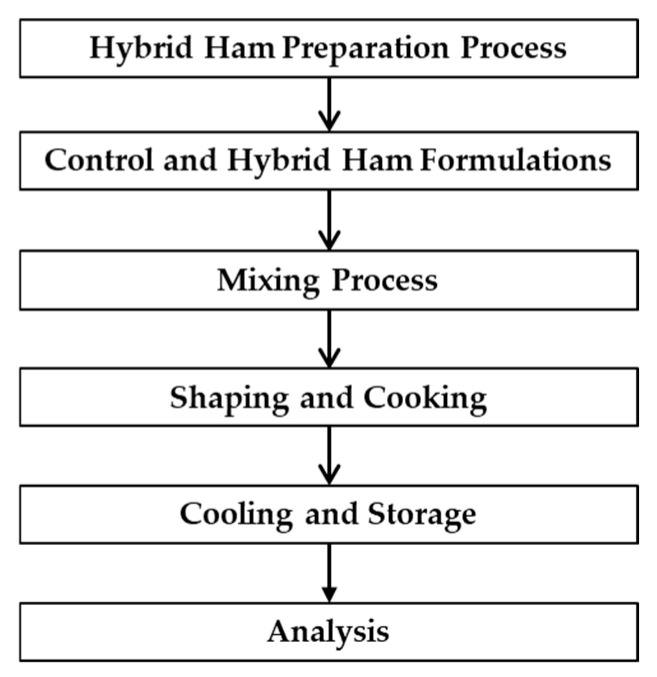
Diagram of the preparation process of the hybrid hams.

The preparation process of the hybrid hams was carried out as shown in Figure 1, where a formulation without the addition of insect powder was first developed (control) using the following ingredients: pork meat, egg white, ice, salt, spices, natural colorant, oil, dextrose, acerola extract, aroma, and a natural ingredient to replace sodium tripolyphosphate. Afterward, three hybrid hams were developed, where 25% of the pork meat was replaced by *Tenebrio molitor* powder (10% *w/w*) (10T), or *Alphitobius diaperinus* (10% *w/w*) (10A), or *Alphitobius diaperinus* plus *Tenebrio molitor* powders (5% *w/w* of each) (10TA).

The ingredients were mixed using a Thermomix TM31 (Bimby, Vorwerk, Germany) following a specific order, with a gradual increase in speed from 4 to 6 over a total duration of 3 min and 30 s. The mixture was then packed in synthetic casing and subjected to a water bath cooking process at 77 °C until the product’s core temperature reached 70 °C (45 min). Subsequently, the products were refrigerated at 4 °C for 24 h prior to analysis.

### 2.2. Color Measurement

The color measurements were performed in triplicate using a Chroma Meter CR 400 Series Konica Minolta (Osaka, Japan), calibrated to white standards prior to measuring. The samples were sliced into 10 mm thickness and then the color was measured at room temperature. The data L*, a*, and b* were analyzed, where L* indicates brightness (values increase from 0 to 100), a* indicates the degree of redness or greenness (+60, red; −60, green), and b* indicates the degree of yellowness or blueness (+60, yellowness; −60, blueness). The comparison between two samples can be obtained via the determination of the total color difference (ΔE) using Equation (1) [23], as follows:

(1) $$\Delta E=\sqrt{({L*}_{i}-{L*}_{0}{)}^{2}+({a*}_{i}-{a*}_{0}{)}^{2}+({b*}_{i}-{b*}_{0}{)}^{2}\;}$$

The letter i indicates the different hybrid hams with insect powder values and 0 is for the values of the control sample. When comparing the samples, the observer detects distinct colors if the ΔE value is more than 5 [24].

### 2.3. Nutritional Composition

Nutritional composition analyses were carried out in triplicate for the hybrid hams, as well as for the powder insect-added ones. The protein content of the samples was determined using the DUMAS method (Thermo Quest NA 2100 Nitrogen and Protein Analyzer, Interscience, Breda, The Netherlands), obtained by multiplying the total nitrogen content by the conversion factor of 6.25 for the hybrid hams [25] and 5.33 for the insect powders [26] to reduce overestimation of protein content due to the presence of chitin. The total fat content was measured according to the Soxhlet extraxtion method with petroleum ether. The extraction was performed in a Soxhlet extractor (Tecator Soxtec System HT 1043 Extraction unit plus Tecator Soxtec System HT 1046 Service unit AB, Hoganas, Sweden). The crude fat was determined gravimetrically, after solvent evaporation in a rotary evaporator and drying in an oven [27].

The moisture content was determined at 105 °C (Binder, GmbH, ED056, Tuttlingen, Germany) until a constant weight was obtained [28]. The total ash content was determined by incineration at 550 °C in a muffle furnace, until the ashes turned white [29]. The carbohydrate content was calculated as the difference to 100 from the sum of protein, lipid, and ash.

The measurement of minerals (Na, K, Ca, Mg, P, S, Fe, Cu, Zn, and Mn) was performed using Inductively Coupled Plasma Optical Emission Spectrometry (ICP-OES) with a Thermo Scientific ICAP Series 7000 (Thermo Fisher Scientific, Waltham, MA, USA) [30]. Approximately 0.5 g of the sample was weighed into tubes, to which 8 mL of 37% hydrochloric acid (HCl) and 2 mL of 65% nitric acid (HNO_3_) were added. The samples were then digested overnight. After digestion, deionized water was added to bring the volume to 50 mL. Finally, the samples were transferred to amber glass vials and analyzed using the optical emission spectrometer.

Amino acid determination was performed according to Machado et al. [31]. To conduct hydrolysis of amino acids, 25 mg of each sample was weighed in triplicate. Each sample was mixed with 5 mL of 6 M hydrochloric acid, sealed and subjected to hydrolysis at 110 °C for 24 h. Once hydrolysis was complete, samples were cooled to room temperature and the pH was adjusted to 2 using NaOH. The hydrolyzed solution was transferred to a 50 mL volumetric flask, followed by the addition of 1 mL of internal standard (L-norvaline, 5 mM). The final volume was adjusted to 50 mL using distilled water to ensure proper dilution based on calibration curves. A 1 mL aliquot was then filtered using a 0.22 µm syringe filter into a centrifuge tube and stored at 4 °C for subsequent analysis.

For the hydrolysis of tyrosine and tryptophan, 25 mg of each sample was accurately weighed in duplicate. Each sample was treated with 5 mL of 5 M NaOH, sealed tightly and subjected to hydrolysis at 120 °C for 12 h. After hydrolysis, the samples were cooled to room temperature, and the pH was adjusted to 2 using 6 M HCl. The solution was then transferred to a 50 mL volumetric flask, and 100 μL of an internal standard (tramadol hydrochloride at 500 μg/mL) was added. The final volume was brought to 50 mL using distilled water. A 500 μL aliquot was filtered through a 0.22 µm syringe filter and transferred into an HPLC vial for analysis.

Chromatographic analysis was performed using a Thermo Scientific Dionex UltiMate 3000 Series system (Thermo Fisher Scientific, USA), which included an RS quaternary pump, a WPS-3000RS autosampler (maintained at 4 °C), a TCC-3000RS column compartment (held at 35 °C), and an FLD-3400RS fluorescence detector, with excitation and emission wavelengths set at 250 nm and 395 nm, respectively.

Amino acid separation was achieved using an ACE 5 C18 column (5 μm, 150 × 4.6 mm i.d.), employing a ternary gradient method. The mobile phase included 140 mM sodium acetate, 17 mM triethylamine, and 1 mM EDTA at pH 4.95 (phase A), along with 60% acetonitrile (phase B) and water (phase C). The gradient program began at 100% phase A, then transitioned to 33% phase B and 7% phase C over 40 min. It then increased to 40% phase B while reducing phase C to 0% over 8 min, before reaching 100% phase B in 0.5 min and maintaining this composition for 5.5 min. The column was re-equilibrated for 10 min before the next injection.

For tyrosine and tryptophan analysis, a different gradient was used with 50 mM NaH_2_PO_4_ (phase A) and acetonitrile (phase B). The gradient started at 5% phase B, increased to 60% over 8 min, was held for 1 min, then returned to initial conditions over 0.5 min, followed by a 3.5 min re-equilibration. The injection volume was set to 5 μL and the column oven was maintained at 40 °C. Fluorescence detection was performed with a dynamic timetable: excitation at 274 nm, emission at 304 nm, shifting to excitation at 280 nm and emission at 340 nm at 3 min, and then to excitation at 202 nm and emission at 296 nm at 5 min.

Data processing and results interpretation were conducted using Chromeleon software (version 7.2, Thermo Fisher Scientific, USA).

### 2.4. Total Phenolic Compounds and Antioxidant Activity Determination

All the analyses of hybrid hams were carried out in triplicate. Firstly, phenolic compounds were extracted using methanol–water (80:20, *v*/*v*), where 10 mL was added with 2 g of sample, and the mixture was shaken overnight. The supernatant was separated by centrifugation at 18,000× *g* at 20 °C for 10 min and a new extraction was performed twice. The supernatant was stored at 4 °C until use. All the analyses were performed in the microplate reader CLARIOstar^®^ Plus (BMG LABTECH GmbH, Ortenberg, Germany).

Total phenolic content (TPC) determination is based on the Al-Duais et al. [32] method, with some modifications, using the reagent Folin-Ciocalteu. In a microplate, 100 μL of Folin reagent was added to 20 μL of the samples. After 5 min, 80 μL of ${Na}_{2}CO_{3}$ was added, and the absorbance was read at 760 nm after 2 h sheltered in the dark. The phenolic content was expressed in gallic acid equivalents per g using a calibration curve of gallic acid.

The supernatant was also used for the antioxidant analysis using 2,2-diphenyl-1 picrylhydrazyl (DPPH) and Ferric Reducing Antioxidant Power Assay (FRAP) methods. For both, a calibration curve was previously prepared using Trolox, and the results were expressed in Trolox equivalents per g. Antioxidant activity analysis using the FRAP method is based on Carrasco-Sandoval et al. [33] method with some modifications. FRAP reagent is produced by mixing 0.5 mL of TPTZ (ferric 2,4,6-tripyridyl-s-triazine), 0.5 mL of FeCl_3_, and 5 mL of acetate buffer and then leaving it for 15 min at 37 °C. In a microplate, 175 μL of the FRAP reagent was added to 25 μL of sample and then placed in the dark for 30 min before measuring the absorbance at 595 nm.

Antioxidant activity analysis using DPPH was performed as described by Herald et al. [34] with some modifications. A total of 20 μL of the sample was added to 180 μL of DPPH solution (150 μmol L^−1^) in methanol–water (80:20, *v/v*) for 60 s in a 96-well microplate. After 40 min in the dark at room temperature, the absorbance was measured at 515 nm.

### 2.5. Texture Profile Analysis (TPA)

The texture of the samples was evaluated using a Texturometer TA-XT plus (Stable Micro Systems, Surrey, United Kingdom) [35]. The samples were cut into 10 mm slices with a SAS 120 C1 electric multipurpose slicer (SilverCrest, TARGA GmbH, Soest, Germany). Puncture tests were conducted with a cylindrical probe (10 mm diameter) to a depth of 4 mm. The samples were compressed at a speed of 1 mm/s using a 5 kg load cell. The TPA was carried out at 20 °C and repeated five times to measure firmness and adhesiveness. Additionally, the cohesiveness of the samples was calculated.

### 2.6. Rheology Measurement

The rheological properties of the ham samples were assessed using a controlled-stress rheometer (Haake Mars III, Thermo Fischer Scientific, Waltham, MA, USA). Initially, a stress sweep was performed to determine the linear viscoelastic region (LVR). Subsequently, the mechanical spectra, represented by the storage modulus (G′) and loss modulus (G″) as functions of frequency, were obtained [36]. The samples were cut into slices with a thickness of 2.5 mm. A serrated parallel plate system with a 20 mm diameter (PP20) and a gap of 2.0 mm between the plates was utilized. The tests were carried out at 20 °C, with the samples protected against dehydration by a lid. Each analysis was conducted at least three times.

### 2.7. Sensory Evaluation

The sensory evaluation was conducted with an untrained panel of 40 participants (19–63 years old; 10 male, 30 female) in individual sensory booths in accordance with ISO 8589:2007 [37]. The tasting panel followed the standardized procedures established by the LEAF research center (Linking Landscape, Environment, Agriculture and Food) at the Instituto Superior de Agronomia, Portugal. Volunteers received informed consent forms in line with the ethical guidelines of the local human experimentation committee and the World Medical Association’s Code of Ethics (Declaration of Helsinki, 1975, revised in 2013).

To assess consumer preference, ham with no insect powder incorporation (control), hybrid ham with 10% of *T. molitor* powder, hybrid ham with 10% of *A. diaperinus* powder, and hybrid ham with 5% of each insect powder were randomly presented to the consumers. All participants were previously informed that the samples contained the powder of edible insects, approved by EFSA.

The samples were evaluated based on overall appearance, texture, aroma, flavor, and overall impression, using a five-level hedonic scale, ranging from “I liked it very much—5 points” to “I dislike it very much—1 point”. Additionally, participants rated their purchase intention on a five-level scale ranging from “I would definitely buy” (5 points) to “I definitely wouldn’t buy” (1 point). The hams were stored at 4 °C before the tasting evaluation. The samples were presented to the participants, and a glass of water was provided to cleanse the palate between samples.

### 2.8. Statistical Analysis

All data were analyzed using GraphPad Prism software (version 5). Analysis of variance (ANOVA), followed by Tukey’s test or *t* test, was performed to compare average values between samples with a 5% significance level (*p* < 0.05).

## 3. Results and Discussion

**Figure 2:**
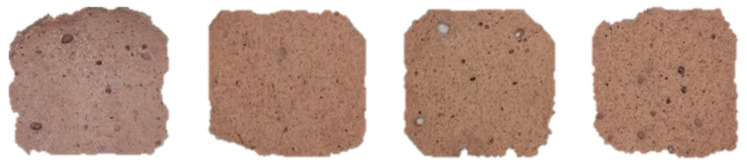
Images from left to right: Control, 10T, 10A, and 10TA hybrid hams.

Based on the results presented in Table 1, significant differences (*p* < 0.05) in the color parameters of the control and hybrid hams can be observed. The control ham, without insect inclusion, exhibited a brighter color (L* = 66.92), a more intense red color (a* = 10.02), and a lower yellowness intensity (b* = 10.89) compared to the hybrid samples. Among the hybrid hams, samples with 10% insect inclusion (both *T. molitor* and *A. diaperinus*) showed the lowest L* and a* values, with a slight rise in b* values, indicating that the presence of insects influenced both lightness and chromaticity of the ham (Figure 2).

When analyzing the ΔE value, which represents the total color difference, it becomes evident that human perception can distinguish between the control and hybrid samples (ΔE > 5) [24]. However, the sample containing 5% of each insect (*T. molitor* and *A. diaperinus*) was closer in overall color to the control, with a ΔE of 6.55. This suggests that a 5% inclusion of each insect powder minimized the perceptible differences from the control ham. These findings emphasize the importance of ingredient proportion in achieving desirable color attributes in hybrid meat products.

These results align with observations from other researchers. In a study involving maize tortillas enriched with *T. molitor* larvae powder (6.5%), the tortillas were found to be darker than the control [38]. Additionally, research on extruded cereal snacks containing varying levels of grasshopper (*Sphenarium purpurascens* Ch.) powder (0–40%) demonstrated that increasing insect proportions led to higher total color differences (ΔE) [39]. Another study analyzed the effect of partially substituting *Tenebrio molitor* powder on biscuit properties and found that the biscuits exhibited a darker color with increasing levels of insect powder substitution [40].

These results are consistent with the findings of this study and reinforce the idea that insect incorporation tends to produce darker products. This highlights the need for strategies to manage the interplay between insect content and visual attributes, emphasizing the importance of optimizing inclusion levels to balance innovation with consumer preferences.

The data presented in Table 2 highlights the impact of insect powder inclusion on the nutritional characteristics of hybrid hams. This inclusion significantly improves the protein, lipid, and mineral content of hybrid hams, while reducing carbohydrate and moisture levels. These findings highlight the potential of *T. molitor* and *A. diaperinus* as sustainable ingredients for use in new foods.

Moisture content decreased with insect inclusion, with the 5% of each insect sample and the 10% *A. diaperinus* sample showing significantly lower values (57%) compared to the control (66%). Some studies support this observation, such as Roncolini et al. [17], who investigated the incorporation of *Tenebrio molitor* and *Alphitobius diaperinus* larvae into cooked sausages, and Kim et al. [6], who examined the effects of substituting meat with *Tenebrio molitor* larvae in meat emulsions. Their findings revealed that the addition of these insect powders resulted in a reduction in moisture content compared to the control samples. This decrease in moisture is attributed to the higher protein content and lower water-binding capacity of insect powders compared to traditional meat proteins, which influences the overall water retention of the final product.

Protein content increases substantially with insect powder addition in hybrid hams, with the highest levels observed in the sample containing 10% *T. molitor* (49%), followed by 10% *A. diaperinus* (46%) and 5% of each insect (40%) powder. This increase aligns with the known nutritional benefits of insects, which contain high protein levels, as may have been verified in this study (38% for *T. molitor* powder and 61% for *A. diaperinus* powder). Also, Kim et al. [6] applied *T. molitor* larvae as an ingredient in sausages, and the results showed that when using untreated *T. molitor* larvae, the protein content was 23%, while after adding 10% of larvae, the content increased to 26%. This finding further supports the results observed in other studies by Kurečka et al. [41], Tang et al. [8], and Wu et al. [42], which highlight the protein-rich nature of edible insects, positioning them as an excellent alternative protein source for food formulations.

The protein content of the hybrid ham sample containing 5% of each insect was expected to fall between the values observed for the samples with 10% *T. molitor* and 10% *A. diaperinus*. This variation may be attributed to specific physicochemical interactions and structural modifications resulting from the combination of insect-derived proteins. Herdeiro et al. [43] provide further evidence supporting this hypothesis, demonstrating that 3D-printed insect-based snacks formulated with *T. molitor* and *A. diaperinus* exhibited non-linear deviations in protein content. These variations were associated with protein interactions, structural rearrangements, and modifications in textural properties. These findings emphasize the complexity of predicting the protein composition of composite insect-based formulations, reinforcing the limitations of linear additive assumptions.

It is important to emphasize that overestimating the protein content in insects intended for human and animal consumption has sparked significant discussions in the scientific literature. Research has demonstrated that chitin, a structural polysaccharide present in the exoskeletons of insects, contributes nitrogen that is not derived from true protein, leading to overstated protein estimates and compromising the accuracy of reported data [44,45].

Boulos et al. [26] examined nitrogen-to-protein conversion factors for edible insects, including *T. molitor*, *A. domesticus,* and *L. migratoria*. Their study found that the conventional nitrogen-to-protein conversion factor of 6.25 frequently overestimates the protein content in insects. They proposed species specific factors, such as 5.33, which was calculated as an average from seven different batches of three insect species. This value provides a more accurate reflection of true protein content compared to the traditional 6.25 factor and is strongly supported by data from the literature, even for insect species belonging to other orders. Addressing this challenge through improved methodologies and greater transparency in scientific reporting is essential for fostering confidence in the use of insects as a sustainable source of protein.

Lipid content also showed an increase, with the highest levels in the 10% *T. molitor* (40%), followed by the 10% *A. diaperinus* and 5% of each insect (37% in both) hybrid hams. This increase in lipid content is consistent with the fat composition of *T. molitor* and *A. diaperinus* powders (29% and 17%, respectively) and corroborates similar observations in a study developed by Jankauskienė et al. [46] about the inclusion of *T. monitor* in pork sausage formulations.

Notably, this elevated lipid content could contribute positively to the sensory attributes, such as flavor and texture, of the hams, which is essential for consumer acceptance [47]. Also, according to Kolobe et al. [48], they could be a source of essential fatty acids, replacing conventional edible oils for human consumption. Kowalski et al. [49] verified that *A. diaperinus* provides significant amounts of palmitic acid (23.76%) and linoleic acid (26.93%), while *T. molitor* contains a high concentration of oleic acid (42.95%).

Incorporating insects into the human diet presents a sustainable and nutrient-dense alternative to traditional fat sources, enhancing dietary diversity and contributing to global food security. However, current research about insects primarily focuses on proteins, highlighting the need for more comprehensive and in-depth studies on insect lipids [50].

In contrast, carbohydrate content decreased with insect inclusion, particularly in the 10% *T. molitor* (2%) and 10% *A. diaperinus* (8%) samples compared to the control (30%). This aligns with the low carbohydrate composition of insect powders, where *T. molitor* contains 30% and *A. diaperinus* contains 19%.

Ash content was also notably higher in insect-enriched samples. The control sample contained 8% ash, whereas insect-enriched hams were around 10%. This trend mirrors findings from other studies, such as Roncolini et al. [17], which emphasize the potential of insect-based formulations to enhance mineral availability in foods, providing functional food advantages.

With the growing consumption of insect-based products worldwide, it is also important to know their nutritional composition in terms of minerals, as they are considered essential elements necessary for the normal metabolic functioning of the human body. Iron is essential for oxygen transport and cellular function. Also, the bioavailability of iron from insects can be comparable to that of beef, making them a viable alternative for addressing iron deficiencies. Also, edible insects are rich in zinc, which supports immune function and enzyme activity. Regarding copper, it can be found in various insects, where it plays a role in iron metabolism [51,52].

Sodium plays a crucial role in imparting flavor to food, preserving it, and regulating bodily functions. However, most people consume excessive amounts of salt. Therefore, it is necessary to adopt salt reduction measures to improve people’s general level of health [53]. Regarding sulfur, it is a vital element that all living things require, being a component of proteins and other bio-organic substances. In addition, potassium is essential to perform many cellular functions, including maintaining intercellular fluid balance, muscle contraction, nerve impulses, and reducing blood pressure [54,55].

Calcium is important for strengthening the skeleton and is essential for blood clotting, muscle contraction, and nerve transmission, while magnesium is essential for cellular homeostasis and organ function, including oxidative phosphorylation, energy production, glycolysis, synthesis of proteins, and nucleic acids [56].

Both insects and meat serve as important sources of essential minerals. The mineral composition of both varies widely, with insects, regardless of species or developmental stage, typically exhibiting higher levels of calcium, zinc, copper, and manganese compared to meat [52,57].

Based on the mineral composition presented in Table 3, it is possible to verify that, in general, *Alphitobius diaperinus* powder has a higher mineral content compared to *Tenebrio molitor* powder. Specifically, *A. diaperinus* powder exhibits higher values of sodium, potassium, calcium, phosphorus, sulfur, copper, and zinc. These results are consistent with those obtained for hybrid hams, where these differences are reflected in the mineral composition of the hybrid hams that contained *A. diaperinus* powder. This suggests that *A. diaperinus* powder could be a superior ingredient in enhancing the mineral profile of hybrid meat products, potentially contributing to improved nutritional quality.

The comparison of mineral content in edible insect powders with previous studies highlights the nutritional value of *Tenebrio molitor* and *Alphitobius diaperinus* as mineral sources. The findings in this study show that the sodium and calcium values in *T. molitor* powder (51.4 mg/100 g and 48.4 mg/100 g, respectively) are comparable to the values reported by Orkusz [57] (53.7 mg/100 g Na and 42.9 mg/100 g Ca).

For *A. diaperinus*, the mineral values observed in this study were similar to the findings of Roncolini et al. [17] for manganese, at 0.8 mg/100 g, iron, at 6.7 mg/100 g, and phosphorus, at 825 mg/100 g. However, the zinc and magnesium values in *A. diaperinus* powder were lower, with 9.8 mg/100 g and 38.3 mg/100 g, respectively, whereas calcium was higher (68.4 mg/100 g). These variations emphasize the influence of environmental and methodological factors on insect mineral composition. Also, differences in substrate composition and the developmental stages of insects at harvest may contribute to these discrepancies.

When analyzing the mineral composition of hybrid hams with insects (Table 3), it can be seen that they contain mainly sodium, potassium, phosphorus, and sulfur, along with smaller amounts of calcium, magnesium, iron, copper, zinc, and manganese. Notably, the inclusion of insect powders significantly enhanced the mineral profile of the hybrid hams. Also, it is important to highlight that claiming these products as a “source of” phosphorus, iron, copper and zinc may be achieved according to Regulation (EC) nº 1924, 2006 [58].

The recommended daily allowances (RDAs) for minerals, according to the European Food Safety Authority (EFSA), state that adults consume less than 2 g of sodium per day [59]. The sodium levels in insect-enriched hams were within an acceptable range for moderate consumption. For potassium, the EFSA recommends an intake of 3.5 g/day for adults [60]. The hybrid hams contribute approximately 5% of the daily requirement.

The EFSA recommends a daily intake of 550 mg of phosphorus [61]; in this respect, the insect-enriched ham formulations provide approximately 30% of the daily requirement. Concerning calcium, the EFSA recommends a daily intake of 2.5 g for adults [62]. Although hybrid hams provide only a small fraction of this requirement, they contribute to the overall dietary intake, especially when combined with other calcium-rich foods.

The EFSA recommends an intake of 11 mg/day of iron for adults and 16 mg/day for premenopausal women [63]. These hybrid hams contribute approximately 19% of the RDA for adults and 13% of it for premenopausal women.

In a study about pork emulsion sausages, Kim et al. [6] also verified that the incorporation of *T. molitor* powder led to an increase in almost all minerals. Notably, zinc levels increased by 89%, while calcium and magnesium doubled and copper increased sixfold. Additionally, iron content increased by 1.5 times.

There is little data available in the literature to verify the impact of adding insects like *T. molitor* and *A. diaperinus* to meat products in terms of mineral content. This highlights the importance of carrying out additional studies to explore the mineral content and potential benefits of incorporating insects into meat products to provide a comprehensive understanding of their nutritional impact [52].

The amino acid profile analysis of *A. diaperinus* and *T. molitor* powders presented in Table 4 provides valuable insights into the nutritional composition of these insect species. It is important to highlight that the differences observed between these results and data from the literature can be attributed to variations in diet, environmental conditions, physiological state of the larvae, and analytical methodologies. These factors play a crucial role in influencing amino acid concentrations in insect-derived protein sources [44,64].

Also, a comparative analysis of the amino acid profiles of *Tenebrio molitor* and *Alphitobius diaperinus* reveals nutritionally significant differences, highlighting the importance of selecting the appropriate protein source for various food applications. Both species exhibit rich amino acid profiles, but variations exist that may impact their nutritional contributions. The results indicate that *A. diaperinus* contains higher concentrations of histidine (5.39 g/100 g vs. 3.76 g/100 g in *T. molitor*), glycine (2.35 g/100 g vs. 1.20 g/100 g), and threonine (1.27 g/100 g vs. 0.69 g/100 g).

Histidine plays a crucial role in immune function and protein synthesis [65]. Glycine is essential for the production of several essential physiological molecules, such as purine nucleotides and glutathione. Additionally, it is a powerful antioxidant [66,67]. Threonine is needed for the synthesis of proteins and is a precursor of glycine [68].

In this study, the histidine concentration in *T. molitor* powder is higher than the values reported by Xie et al. [40] (2.41 g/100 g) and Jankauskienė et al. [69], with histidine levels ranging from 1.49 to 1.81 g/100 g in lyophilized larvae fed with different diets. This difference in histidine content further demonstrates the influence of feeding regimens on amino acid profiles.

Another notable difference is the significantly higher amount of tyrosine in *A. diaperinus* (115.54 g/100 g) compared to *T. molitor* (88.61 g/100 g). Tyrosine is an essential precursor for dopamine, a key neurotransmitter involved in cognitive function and mental health. Therefore, increasing dietary tyrosine intake can enhance working memory and cognitive performance, particularly in elderly individuals [70].

On the other hand, *T. molitor* has a slightly higher content of aspartic acid + asparagine (8.32 g/100 g vs. 7.54 g/100 g in *A. diaperinus*), alanine (2.96 g/100 g vs. 2.66 g/100 g), and arginine (6.36 g/100 g vs. 5.95 g/100 g). The levels of arginine, which is important in immune function and protein synthesis, for *A. diaperinus* align with those reported by Roncolini et al. [17] (6.07 g/100 g). However, the arginine concentration in *T. molitor* powder was higher than the value reported by Xie et al. [40] (2.75 g/100 g), emphasizing the impact of diet and environmental conditions on amino acid composition.

The protein from *A. diaperinus* and *T. molitor* contains a high nutritional value, with essential amino like histidine, valine, and threonine. The results obtained in this study reinforce the potential of insect-based protein sources as viable alternatives to conventional animal proteins. Additionally, combining these protein sources could be an effective strategy for creating products with more balanced and functional protein profiles. By incorporating *A. diaperinus* and *T. molitor* into food formulations, it is possible to enhance the nutritional quality of foods while contributing to sustainability efforts in protein production.

**Table 4:** Amino acid profile of *A. diaperinus* and *T. molitor* powders compared to findings from other researchers.

Amino Acid (g/100 g)	*T. molitor* Powder	*A. diaperinus* Powder	T. molitor */**	A. diaperinus ***/****
Asp + Asn	8.32 ± 0.16 ^a^	7.54 ± 0.10 ^b^	nd/nd	nd/5.42
Ser	2.62 ± 0.11 ^a^	2.35 ± 0.14 ^a^	2.17/1.39–1.73	4.76/2.84
Glu + Gln	9.01 ± 0.21 ^a^	9.01 ± 0.02 ^a^	nd/nd	nd/7.74
His	3.76 ± 0.19 ^b^	5.39 ± 0.32 ^a^	2.41/1.49–1.81	7.38/2.40
Gly	1.20 ± 0.01 ^b^	2.35 ± 0.10 ^a^	2.53/2.41–2.66	4.62/2.81
Arg	6.36 ± 0.09 ^a^	5.95 ± 0.37 ^a^	2.75/nd	6.07/3.63
Thr	0.69 ± 0.02 ^b^	1.27 ± 0.08 ^a^	2.17/1.27–1.45	4.45/2.58
Ala	2.96 ± 0.10 ^a^	2.66 ± 0.02 ^b^	5.55/3.35–3.74	6.62/4.43
Pro	2.64 ± 0.11 ^a^	2.75 ± 0.13 ^a^	3.79/2.67–2.98	6.68/3.94
Val	2.52 ± 0.15 ^b^	2.87 ± 0.01 ^a^	3.21/2.25–3.14	6.14/3.61
Tyr	88.61 ± 1.22 ^b^	115.54 ± 1.76 ^a^	nd/3.25–3.88	7.89/4.57
Trp	2.33 ± 0.02 ^b^	2.98 ± 0.00 ^a^	nd/nd	nd/1.22

Dry basis. Different letters in the same row indicate significantly different results (*p* < 0.05) according to *t* tests. nd = not determined. * Xie et al. [40], ** Jankauskienė et al. [69], *** Roncolini et al. [17], **** Fuso et al. [71].

According to the results presented in Table 5, the incorporation of insect powders has led to an enrichment of almost all the amino acids presented in the hybrid hams, especially the essential threonine, valine, and histidine, and nonessential serine, arginine, and proline. The inclusion of 10% *A. diaperinus* powder and 5% of each insect powder in the hybrid hams resulted in a significant increase in threonine (1.71 g/100 g and 0.91 g/100 g, respectively) and valine (1.73 g/100 g and 1.76 g/100 g, respectively) compared to the control (0.60 g/100 g and 1.40 g/100 g, respectively). Essential amino acids are indispensable for protein synthesis, aiding in the growth, repair, and maintenance of body tissues. They are also pivotal in various metabolic and physiological processes, such as enzyme and hormone production, immune function, and nutrient absorption [72].

Regarding histidine, it can be observed that although *A. diaperinus* powder presented a greater quantity (5.39 g/100 g) compared to *T. molitor* powder (3.76 g/100 g) (Table 4), this was not reflected in its incorporation into the ham (Table 5), as there was no significant difference between the hams with the addition of insect powder. This discrepancy could be attributed to protein matrix interactions or differences in processing (i.e., extremes of temperature and pH), where under these conditions some amino acid sidechains are susceptible to undergoing chemical reactions [73,74]. However, histidine, which has immunological benefits [65], was markedly higher in the 10% *A. diaperinus* and 5% of each insect formulation (3.13 g/100 g and 2.94 g/100 g, respectively) compared to the control (2.47 g/100 g).

The addition of insect powders caused an increase in serine from 1.24 g/100 g (control) to 1.38 g/100 g up to 1.49 g/100 g. Arginine, which supports immune function and cardiovascular health [65,75], showed a similar trend, increasing from 4.83 g/100 g (control) to 5.57 g/100 g (5% of each insect formulation). Conversely, glycine and alanine levels in the hybrid hams remained similar to those in the control formulation, suggesting that these amino acids are less affected by insect protein incorporation.

The study developed by Cho and Ryu [76] investigated the effects of different *Tenebrio molitor* contents and extrusion process parameters on the physicochemical properties of the extruded meat analogue. The results indicated that the total amino acid content increased after the addition of *T. molitor* larvae; however, subsequent denaturation due to high temperature during extrusion led to a decrease. After extrusion, glutamic acid, a flavor-enhancing amino acid, was found to be the most abundant, with the highest concentration observed in the meat analog containing 30% *T. molitor* larvae.

Jankauskienė et al. [46] conducted a study incorporating various proportions of *Tenebrio molitor* (10%, 20%, and 30%) into sausage formulations. They observed that this inclusion positively impacted the amino acid quality, with the most favorable results obtained using 10%. Similarly, the present findings demonstrate that incorporating *T. molitor* powder into hybrid ham significantly enhances the amino acid profile, suggesting its potential as a viable alternative to conventional meat products.

Specifically, the serine, histidine, arginine, and valine contents were higher in hybrid hams compared to sausage (0.79 g/100 g, 0.18 g/100 g, 0.92 g/100 g, and 0.40 g/100 g, respectively). Glycine (1.00 g/100 g) and alanine (1.07 g/100 g) levels in sausage [46] were similar to hybrid hams, indicating that certain amino acids were retained similarly despite differences in processing conditions.

In this study, the inclusion of *T. molitor* and *A. diaperinus* powders in hybrid hams resulted in an overall improvement in amino acid composition. Although specific data on the incorporation of *A. diaperinus* in meat and meat analogues are limited, the potential of these insect proteins for such applications deserves further investigation. Insects, including *A. diaperinus*, are rich in high-quality proteins and essential amino acids, making them a promising alternative for developing meat analogues that closely mimic the nutritional and sensory properties of conventional meat.

Additionally, studies have highlighted the benefits of incorporating edible insect ingredients in meat-type products. For instance, Choi et al. [77] demonstrated that replacing lean pork meat with up to 10% *T. molitor* successfully maintained the quality of frankfurters at a level similar to that of regular control frankfurters. Moreover, Kim et al. [78] concluded that up to 40% of pork myofibrillar protein could be replaced by *T. molitor* protein in emulsion systems. Pasqualin et al. [79] indicated that incorporating *Acheta domesticus* as a lean meat replacer in beef patties in quantities up to 5% maintained optimal quality. Furthermore, Gomes et al. [80] reported that hybrid beef patties with *Gryllus assimilis* replacing beef for up to 7% of the formulation achieved adequate technological and sensory characteristics.

These findings underscore the sustainability and nutritional benefits of insects, making them valuable functional ingredients in the evolving landscape of alternative proteins. The successful incorporation of insect powders in hybrid hams and other meat-type products supports their potential as sustainable and nutritious alternatives in the food industry. Edible insects clearly hold significant promise for creating innovative and sustainable protein sources. However, further research is necessary to fully explore their potential and optimize their integration into mainstream food products.

Insect incorporation also improved the antioxidant properties of the hybrid hams, as evidenced in Table 6. The control hybrid ham exhibited a TPC of 0.37 mgGAE/g, which increased significantly in samples containing 10% *A. diaperinus* (0.70 mgGAE/g) and the combination of 5% *T. molitor* and 5% *A. diaperinus* (0.69 mgGAE/g). FRAP values followed a similar trend, with the highest antioxidant activity observed in the 10% *A. diaperinus* sample (0.22 mgTE/g). However, for DPPH values, no significant differences between formulations were found, suggesting that certain antioxidant mechanisms might be more dominant depending on the assay.

Antioxidants are a group of compounds that work to inhibit or mitigate the harmful effects caused by free radicals and other reactive nonradical species [81,82]. However, it is important to note that measuring the antioxidant capacity of a compound using a single method is challenging. This is because antioxidant mechanisms in biological matrices are highly complex, involving numerous interacting factors [83]. For this reason, two different methodologies (DPPH and FRAP), each based on distinct antioxidant mechanisms, were employed in this study. The DPPH assay is based on the ability of antioxidants to act as hydrogen donors, reducing the stable 2,2-diphenyl-1-picrylhydrazyl (DPPH) radical to its non-radical, yellow-colored diphenyl-picrylhydrazine (DPPH-H) form [81]. Regarding the Ferric Reducing Antioxidant Power (FRAP) assay, it operates on the principle of electron transfer, where antioxidants act as reducing agents by donating electrons to the Fe^3^⁺-TPTZ complex. This reduction converts the initially colorless Fe^3^⁺-TPTZ complex into its intensely blue Fe^2^⁺-TPTZ form [84].

In a study developed by Herdeiro et al. [43] to analyze the nutritional properties of snacks containing *T. molitor* and *A. diaperinus*, it was demonstrated that TPC and antioxidant activity were present in all formulations, including the control (0.11 gGAE/g and 0.27 AAE mg/g, respectively). However, snacks containing insect powders exhibited significantly higher TPC (0.59 to 0.64 gGAE/g) and antioxidant activity (0.75 to 0.99 AAE mg/g) values.

Phenolics are renowned for their ability to neutralize free radicals and chelate prooxidant metals, providing oxidative stability and potential health benefits, as observed in studies of insects like *T. molitor* and other species. Insects are known to possess bioactive compounds, including phenolic compounds derived from their diets or synthesized through sclerotization. This is the process by which the insect cuticle hardens through the incorporation of phenolic compounds into the cuticular matrix. This process involves structural proteins and chitin, facilitated by a series of enzyme-mediated reactions [85].

It is also important to highlight that the control formula exhibits a total phenolic content (TPC) when measured using the F-C method. While phenolics are not typically expected in meat, however F-C interferents such as tyrosine and other compounds like organic acids can be present [86,87].

The significant increase in TPC observed in hams containing *A. diaperinus* suggests that this insect contributes more phenolic compounds compared to *T. molitor*. Research by Navarro del Hierro et al. [88] indicates that *T. molitor* larvae possess measurable levels of phenolic compounds, which are associated with their antioxidant activities. However, specific data on the phenolic content of *A. diaperinus* are limited. Nevertheless, the findings obtained from this study highlight the potential of *A. diaperinus* as a functional ingredient to improve the nutritional and preservative qualities of meat products. Further research is warranted to quantify the specific phenolic compounds in *A. diaperinus* and to understand their bioavailability and health implications.

In general, these results along with findings from Herdeiro et al. [43], reinforcing the role of edible insects as a source of bioactive compounds, particularly phenolic compounds, which contribute to enhanced antioxidant activity. This suggests that incorporating edible insects, such as *T. molitor* and *A. diaperinus*, not only enhances the nutritional value of the foods but also improves their bioactive profile. This aligns with sustainability goals and consumer trends favoring functional foods with added health benefits.

It is worth highlighting that other compounds, such as alkaloids, terpenoids, amino acids, and fatty acids, also contribute significantly to the antioxidant effects in insect-based biomass. Maciejewska et al. [15] investigated the functional properties of *Tenebrio molitor* protein hydrolysates, particularly focusing on their antioxidant activity, and found that the hydrolysis process significantly enhances antioxidant activity. Sousa et al. [20] demonstrated that *Alphitobius diaperinus* protein hydrolysates exhibit relevant antioxidant activity. Additionally, Son et al. [89] study indicated that lipids from *Tenebrio molitor* larvae are rich in bioactive nutrients, especially γ-tocopherol, a natural antioxidant that prevents the oxidation of free radicals and hydroperoxy radicals in fat-soluble foods. These findings emphasize the nature of insect-based biomass as a source of various antioxidant compounds, underscoring their potential as valuable functional ingredients in developing sustainable and nutritious food products.

**Figure 3:**
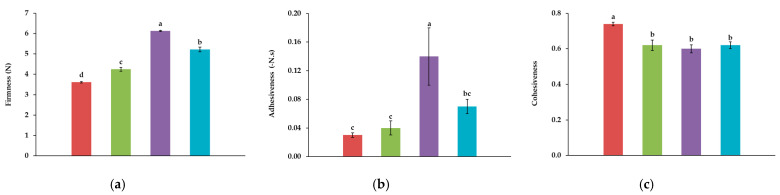
Texture Profile Analysis of Firmness (**a**), Adhesiveness (**b**) and Cohesiveness (**c**) for hybrid hams: 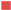 Control, 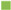 10T, 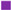 10A, and 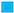 10TA. Different letters indicate significantly different results (*p* < 0.05) according to the Tukey test.

Figure 3 presents the results of the Texture Profile Analysis for the hybrid ham samples, showing that the incorporation of insect powder significantly modifies textural properties. Higher firmness and adhesiveness in *A. diaperinus* formulations could be due to enhanced protein binding. The combined formulation (5% *T. molitor* and 5% *A. diaperinus*) exhibits intermediate behavior, suggesting a balancing effect of the two insect types.

The observed changes in texture properties can also be connected to the substantial increase in protein content with the addition of insect powder. The highest protein content was observed in the hybrid ham containing 10% *T. molitor* (49%). This increase is consistent with the known high protein levels of insect powders, reported in this study as 38% for *T. molitor* and 61% for *A. diaperinus.* The high protein content not only enhances the nutritional profile but also contributes to texture modifications, as proteins are essential to forming a robust gel network, which imparts firmness to food products [25,76,90].

These results align with findings from previous studies. For example, texture hardness values in sausages with *T. molitor* additions have been associated with the protein content and structure of this insect, as demonstrated by Jankauskienė et al. [46]. Similarly, Kim et al. [6] demonstrated that pre-treated *T. molitor* larvae in emulsion sausages enhanced hardness, supporting the conclusion that insect protein contributes to increased textural rigidity. These findings corroborate our results, where insect-enriched formulations showed significant increases in firmness, underscoring the structural impact of insect-derived proteins.

In another study, developed by Aybar et al. [91], *T. molitor* powder was tested as a clean label ingredient in commercial hummus sauce, examining its impact on rheology and texture properties, as well as on microstructure. Sauces containing up to 7.5% *T. molitor* powder maintained their structure, while higher insect concentrations resulted in significant structural and textural changes. The nutritional profile also improved, with increased protein, minerals, and antioxidant capacity.

The control sample exhibits significantly higher cohesiveness compared to the insect-enriched formulations, with no significant differences among them. This suggests that while insect powders enhance other texture properties like firmness, their inclusion might disrupt the natural cohesion of the meat matrix. The reduction in cohesiveness may be attributed to the dilution effect caused by adding non-meat components, such as insect powders, as reported in other research. For example, Cho and Ryu [76], found that adding 15% and 30% of *T. molitor* powder to extruded meat analogues resulted in a reduction in cohesiveness. For snacks containing insect powders, Herdeiro et al. [43] noted similar trends, where structural improvements in firmness and elasticity were offset by slight reductions in cohesiveness.

**Figure 4:**
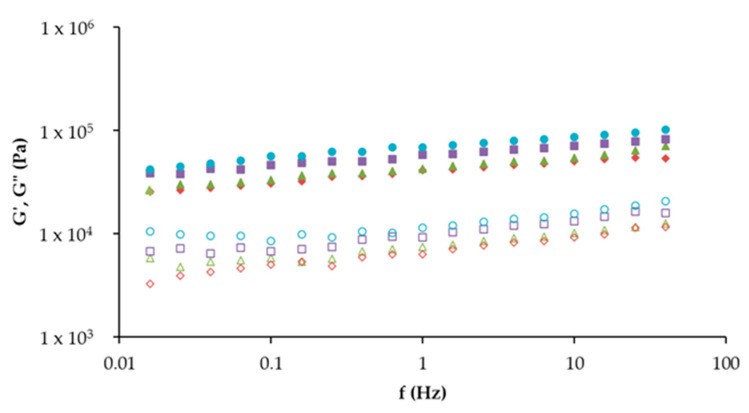
Mechanical spectra of hybrid hams: 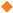 Control, 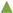 10T, 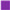 10A, and 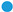 10TA. G′ (full symbol) corresponds to the storage moduli and G″ (open symbol) corresponds to the loss moduli.

Regarding Figure 4, the mechanical spectra were similar in all cases, with elastic modulus (G′) being higher than viscous modulus (G″), indicating that the samples exhibit predominantly elastic behavior. Based on these results, the incorporation of insect powders significantly enhances the viscoelastic properties of hybrid hams. The control sample demonstrated the lowest G′ value, reflecting a softer and less elastic structure compared to the insect-enriched formulations. It is possible to verify that the products containing 10% *A. diaperinus* and 5% *T. molitor* and 5% *A. diaperinus* were those that presented the highest degree of structuring, where the improved G′ and G″ values reflect a stronger protein matrix. This corroborates with the texture results and is due to the higher protein content of *A. diaperinus* powder (61%).

The sample with a combination of 5% *T. molitor* and 5% *A. diaperinus* exhibited intermediate G′ and G″ values. This suggests a synergistic interaction between the two insect powders, where their combined properties balance their structural and textural contributions.

The increase in G′ across insect-enriched samples demonstrates the potential for insect powders to act as structuring agents in hybrid hams, improving their mechanical strength and textural properties. This is consistent with studies such as that by Roncolini et al. [17], where insect powders were shown to improve the structural integrity of baked products.

**Figure 5:**
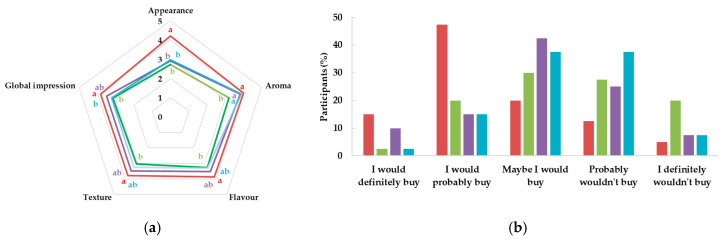
Sensory scores (**a**) and purchase intention (**b**) of hybrid hams: 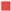 Control, 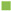 10T, 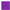 10A, and 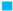 10TA. Different letters indicate significantly different results (*p* < 0.05) according to the Tukey test.

The sensory evaluation results (Figure 5) indicate that the hybrid ham formulations with insect powders were well-received in terms of overall appearance, flavor, and texture, especially the sample of 10% *A. diaperinus* and the combination of 5% *T. molitor* and 5% *A. diaperinus*. Regarding the sensory evaluation of texture, the increased firmness observed in *A. diaperinus* formulations, as well as in the combined formulation (5% *T. molitor* and 5% *A. diaperinus*), as shown in Figure 3, may have contributed to higher sensory acceptance.

Among the samples, the combination of the two insects showed intermediate sensory scores, suggesting that blending these powders can mitigate potential off-flavors or textural challenges associated with individual insect types. Previous research supports this synergistic effect; Herdeiro et al. [43] found that combining these insect powders in 3D-printed snacks improved sensory appeal while enhancing nutritional profiles.

Regarding consumer purchase intention, the control sample had the highest purchase intent and the 10% *A. diaperinus* formulation demonstrated comparable acceptance, indicating potential market viability. Similar findings were reported by Roncolini et al. [17], who noted that partial substitution with *A. diaperinus* powder in baked products maintained consumer acceptability while significantly improving protein content and functional properties.

Additionally, the integration of edible insects into hybrid hams aligns with findings from research conducted by Krawczyk et al. [92], which demonstrated that edible insects could be successfully incorporated into plant-based burger analogues without significantly affecting the physicochemical attributes of the products. Sensory analysis confirmed that their inclusion maintained acceptability, which is particularly valuable for developing insect-enriched foods in regions unfamiliar with entomophagy. These findings underscore the versatility and potential of insect proteins in expanding their use across diverse food systems.

The positive reception of hybrid hams enriched with edible insect powders underscores their potential in new food development. However, the balance of sensory characteristics remains critical for consumer acceptance, as higher inclusion levels may introduce off-flavors or excessive firmness. These findings align with broader trends in entomophagy research, which emphasize the need for innovative formulations to improve palatability while leveraging the high nutritional value of insects. Educating consumers about the sustainability and health benefits of insect-based products may further increase their market acceptance [93,94].

The results obtained in this study highlight that the incorporation of insect-based protein ingredients into food formulations has the potential to enhance the overall nutritional value of meat products. Through this study, it was possible to verify the importance of further research to evaluate the incorporation of insects in meat analogue products, examining their nutritional contributions and technological properties. Additionally, consumer acceptance studies and product development initiatives will be pivotal in integrating insect-based proteins into mainstream food markets, thereby promoting their benefits as sustainable and nutritious food sources.

## 4. Conclusions

This study successfully incorporated *Tenebrio molitor* and *Alphitobius diaperinus* powders into hybrid hams, enhancing protein and mineral content, amino acid profiles, and antioxidant properties. The addition of insect powder influenced physicochemical properties, resulting in a darker color and increased firmness without compromising consumer acceptance. Sensory analysis indicated high acceptance, particularly for formulations with *A. diaperinus* and the combined insect powders, highlighting the potential of insect proteins in processed meat products.

Incorporating insect-based ingredients into processed meat offers a promising strategy for enhancing sustainability and nutritional value in food production. As interest in alternative proteins grows, edible insects present an innovative and environmentally friendly solution for future food formulations.

## Figures and Tables

**Table 1:** Color parameters of the samples: Control, 10T, 10A, and 10TA hybrid hams.

Parameters	Control	10T	10A	10TA
L*	66.92 ± 0.479 ^a^	59.77 ± 0.180 ^c^	60.15 ± 0.363 ^c^	61.27 ± 0.084 ^b^
a*	10.02 ± 0.235 ^a^	7.70 ± 0.025 ^b^	6.49 ± 0.121 ^c^	6.89 ± 0.060 ^c^
b*	10.89 ± 0.238 ^b^	12.28 ± 0.568 ^a^	12.42 ± 0.329 ^a^	11.90 ± 0.112 ^a^
∆E	-	7.65	7.79	6.55

Different letters in the same row indicate significantly different results (*p* < 0.05) according to the Tukey test.

**Table 2:** Nutritional composition of *T. molitor* and *A. diaperinus* powders and of Control, 10T, 10A, and 10TA hybrid hams.

Parameters (%)	*T. molitor* Powder	*A. diaperinus* Powder	Control	10T	10A	10TA
Protein	37.9 ± 1.74 ^B^	61.1 ± 0.68 ^A^	34.9 ± 0.83 ^d^	48.9 ± 1.42 ^a^	46.3 ± 0.67 ^b^	40.4 ± 1.93 ^c^
Lipids	29.1 ± 0.13 ^A^	16.6 ± 0.28 ^B^	24.2 ± 0.75 ^c^	40.1 ± 0.08 ^a^	36.6 ± 0.09 ^b^	36.8 ± 1.74 ^b^
Carbohydrates	30.3	19.2	30.1	1.6	7.5	13.2
Ash	2.7 ± 0.06 ^B^	3.2 ± 0.07 ^A^	7.8 ± 0.39 ^b^	9.5 ± 0.03 ^a^	9.6 ± 0.09 ^a^	9.6 ± 0.20 ^a^
Moisture	5.1 ± 0.12 ^A^	4.1 ± 0.05 ^B^	66.1 ± 0.02 ^a^	58.4 ± 0.11 ^b^	57.3 ± 0.31 ^c^	57.5 ± 0.11 ^c^

Dry basis. Different capital letters in the same row indicate significantly different results for powders according to the *t* test, and small letters stand for hams (*p* < 0.05) according to the Tukey test.

**Table 3:** Mineral composition of *T. molitor* and *A. diaperinus* powders and of Control, 10T, 10A, and 10TA hybrid hams.

Minerals (mg/100 g)	*T. molitor* Powder	*A. diaperinus* Powder	Control	10T	10A	10TA
Na	51.4 ± 1.45 ^B^	95.2 ± 0.32 ^A^	189.8 ± 0.44 ^c^	212.6 ± 4.53 ^b^	221.1 ± 3.78 ^a^	225.0 ± 1.88 ^a^
K	778.7 ± 5.70 ^B^	1003.5 ± 8.70 ^A^	198.3 ± 0.86 ^d^	229.1 ± 1.24 ^c^	253.1 ± 0.50 ^a^	237.4 ± 0.17 ^b^
Ca	48.4 ± 1.09 ^B^	55.6 ± 0.72 ^A^	9.0 ± 0.01 ^c^	10.7 ± 0.57 ^b^	12.7 ± 0.74 ^a^	11.8 ± 0.63 ^ab^
Mg	313.9 ± 0.33 ^A^	186.0 ± 2.71 ^B^	18.9 ± 0.29 ^d^	35.0 ± 0.59 ^a^	25.7 ± 0.30 ^c^	30.1 ± 0.80 ^b^
P	754.7 ± 2.74 ^B^	857.7 ± 10.9 ^A^	**106.5** ** ± ** **0.69 ^c^**	**148.6** ** ± ** **5.52 ^b^**	**164.5** ** ± ** **2.72 ^a^**	**151.0** ** ± ** **1.04 ^b^**
S	318.7 ± 0.58 ^B^	488.33 ± 7.48 ^A^	157.6 ± 1.14 ^d^	162.1 ± 0.05 ^c^	184.5 ± 2.71 ^a^	169.7 ± 0.26 ^b^
Fe	6.2 ± 0.27 ^A^	5.1 ± 0.01 ^B^	1.2 ± 0.01 ^b^	**2.1** ** ± ** **0.03 ^a^**	**2.1** ** ± ** **0.08 ^a^**	**2.1** ** ± ** **0.04 ^a^**
Cu	1.7 ± 0.04 ^B^	2.1 ± 0.07 ^A^	0.0 ± 0.00 ^b^	**0.2 ** ** ±** **0.01 ^a^**	**0.2 ** ** ±** **0.00 ^a^**	**0.2 ** ** ±** **0.00 ^a^**
Zn	13.7 ± 0.48 ^B^	22.9 ± 0.00 ^A^	0.9 ± 0.03 ^d^	**1.7** ** ± ** **0.07 ^c^**	**2.6** ** ± ** **0.05 ^a^**	**2.2** ** ± ** **0.07 ^b^**
Mn	1.1 ± 0.02 ^A^	0.7 ± 0.05 ^B^	0.0 ± 0.00 ^b^	0.1 ± 0.01 ^a^	0.1 ± 0.00 ^a^	0.1 ± 0.01 ^a^

Different capital letters in the same row indicate significantly different results for powders according to the *t* test; small letters stand for hams (*p* < 0.05) according to the Tukey test. The values in bold represent “source of” according to Regulation (EC) nº 1924, 2006 [58].

**Table 5:** Amino acids profile of Control and 10T, 10A and 10TA hybrid hams.

Amino Acid (g/100 g)	Control	10T	10A	10TA
Asp + Asn	5.04 ± 0.59 ^a^	5.44 ± 0.18 ^a^	5.64 ± 0.18 ^a^	5.65 ± 0.37 ^a^
Ser	1.24 ± 0.03 ^b^	1.38 ± 0.09 ^ab^	1.49 ± 0.01 ^a^	1.47 ± 0.03 ^a^
Glu + Gln	7.42 ± 0.43 ^a^	7.27 ± 0.18 ^a^	7.56 ± 0.17 ^a^	7.60 ± 0.24 ^a^
His	2.47 ± 0.06 ^b^	2.55 ± 0.15 ^ab^	3.13 ± 0.09 ^a^	2.94 ± 0.26 ^ab^
Gly	0.84 ± 0.02 ^ab^	0.76 ± 0.07 ^b^	1.10 ± 0.06 ^a^	1.06 ± 0.11 ^a^
Arg	4.83 ± 0.23 ^b^	5.11 ± 0.01 ^ab^	5.56 ± 0.03 ^a^	5.57 ± 0.10 ^a^
Thr	0.60 ± 0.02 ^c^	0.62 ± 0.01 ^c^	1.71 ± 0.28 ^a^	0.91 ± 0.07 ^bc^
Ala	1.60 ± 0.03 ^a^	1.67 ± 0.07 ^a^	1.74 ± 0.04 ^a^	1.73 ± 0.05 ^a^
Pro	0.81 ± 0.03 ^b^	1.07 ± 0.03 ^a^	1.14 ± 0.03 ^a^	1.16 ± 0.05 ^a^
Val	1.40 ± 0.03 ^b^	1.59 ± 0.02 ^ab^	1.73 ± 0.04 ^a^	1.76 ± 0.10 ^a^
Tyr	26.49 ± 0.88 ^c^	39.31 ± 2.06 ^ab^	43.85 ± 4.08 ^a^	33.96 ± 0.00 ^bc^
Trp	1.51 ± 0.02 ^ab^	1.48 ± 0.13 ^ab^	1.67 ± 0.10 ^a^	1.22 ± 0.09 ^b^

Dry basis. Different letters in the same row indicate significantly different results (*p* < 0.05) according to the Tukey test.

**Table 6:** Total phenolic content (TPC) and antioxidant activity (FRAP and DPPH) for the Control, 10T, 10A, and 10TA hybrid hams.

Hybrid Hams	TPC (mgGAE/g)	FRAP (mgTE/g)	DPPH (mgTE/g)
Control	0.37 ± 0.02 ^b^	0.20 ± 0.00 ^b^	0.14 ± 0.00 ^a^
10T	0.39 ± 0.02 ^b^	0.16 ± 0.00 ^c^	0.14 ± 0.01 ^a^
10A	0.70 ± 0.03 ^a^	0.22 ± 0.00 ^a^	0.15 ± 0.00 ^a^
10TA	0.69 ± 0.03 ^a^	0.20 ± 0.02 ^b^	0.15 ± 0.00 ^a^

Different letters in the same column indicate significantly different results (*p* < 0.05) according to the Tukey test.

## Data Availability

The original contributions presented in the study are included in the article; further inquiries can be directed to the corresponding author.
